# Different conformations and packing motifs in the crystal structures of four thio­phene–carbohydrazide–pyridine derivatives

**DOI:** 10.1107/S2056989022005151

**Published:** 2022-05-17

**Authors:** Jennifer L. Garbutt, Cristiane F. da Costa, Marcus V. N. deSouza, Solange M. S. V. Wardell, James L. Wardell, William T. A. Harrison

**Affiliations:** a Department of Chemistry, University of Aberdeen, Meston Walk, Aberdeen AB24 3UE, Scotland; b Fundação Oswaldo Cruz, Instituto de Tecnologia em Fármacos-Far, Manguinhos, 21041-250, Rio de Janeiro, RJ, Brazil; c CHEMSOL, 1 Harcourt Road, Aberdeen AB15 5NY, Scotland; Purdue University, USA

**Keywords:** crystal structure, thio­phene, carbohydrazide, pyridine

## Abstract

The crystal structures of four thio­phene–carbohydrazide–pyridine derivatives, *viz. N*′-[(*E*)-pyridin-3-yl­methyl­idene]thio­phene-2-carbohydrazide, C_11_H_9_N_3_OS, (I), *N*′-[(*E*)-pyridin-2-yl­methyl­idene]thio­phene-2-carbohydrazide, C_11_H_9_N_3_OS, (II), *N*-methyl-*N*′-[(*E*)-pyridin-2-yl­methyl­idene]thio­phene-2-carbohydrazide, C_12_H_11_N_3_OS, (III) and *N*′-[(*E*)-pyridin-2-yl­methyl­idene]-2-(thio­phen-2-yl)ethano­hydrazide, C_12_H_11_N_3_OS, (IV) are described. The dihedral angles between the thio­phene ring and the pyridine ring are 21.4 (2), 15.42 (14), 4.97 (8) and 83.52 (13)° for (I)–(IV), respectively. The thio­phene ring in (IV) is disordered over two orientations in a 0.851 (2):0.149 (2) ratio. Key features of the packing include N—H⋯N_p_ (p = pyridine) hydrogen bonds in (I), which generate *C*(7) chains propagating in the [001] direction; N—H⋯N_p_ links also feature in (II), but in this case they lead to *C*(6) [001] chains; in (IV), classical amide (*C*4) N—H⋯O links result in [010] chains; in every case adjacent mol­ecules in the chains are related by 2_1_ screw axes. There are no classical hydrogen bonds in the extended structure of (III). Various weak C—H⋯*X* (*X* = O, N, S) inter­actions occur in each structure, but no aromatic π–π stacking is evident. The Hirshfeld surfaces and fingerprint plots for (I)–(IV) are compared.

## Abstract 2

The title compounds show different packing motifs including chains mediated by N—H⋯N and N—H⋯O hydrogen bonds.

## 1. Chemical context

Various thio­phene–carbohydrazide derivatives containing a T—C(=O)—NH—N=CH—R (T = thio­phene ring) building unit have been previously investigated by some of us for their anti-cancer (Cardoso *et al.*, 2017 ▸) and anti-tuberculosis (Cardoso *et al.*, 2014 ▸, 2016*a* ▸) properties. Other workers have reported their analgesic activities (Lima *et al.*, 2000 ▸) and their potential uses as tunable photo switches (van Dijken *et al.*, 2015 ▸). The use of these compounds as multi-dentate chelating ligands has been described by Gholivand *et al.* (2016 ▸) and Abbas *et al.* (2021 ▸).

In a continuation of our earlier work on this family of compounds (Cardoso *et al.*, 2016*b* ▸,*c* ▸), we now describe the crystal structures and Hirshfeld surfaces of *N*′-[(*E*)-pyridin-3-yl­methyl­idene]thio­phene-2-carbohydrazide, C_11_H_9_N_3_OS (I), *N*′-[(*E*)-pyridin-2-yl­methyl­idene]thio­phene-2-carbohydrazide, C_11_H_9_N_3_OS (II), *N*-methyl-*N*′-[(*E*)-pyridin-2-yl­methyl­idene]thio­phene-2-carbohydrazide, C_12_H_11_N_3_OS (III) and *N*′-[(*E*)-pyridin-2-yl­methyl­idene]-2-(thio­phen-2-yl)ethano­hydrazide, C_12_H_11_N_3_OS (IV). Compounds (I) and (II) are positional isomers, differing in the location of the N atom of the pyridine ring, (III) is a methyl­ated derivative of (II) and (IV) has a methyl­ene group inserted between the thio­phene ring and the carboyhdrazide grouping compared to (I). 
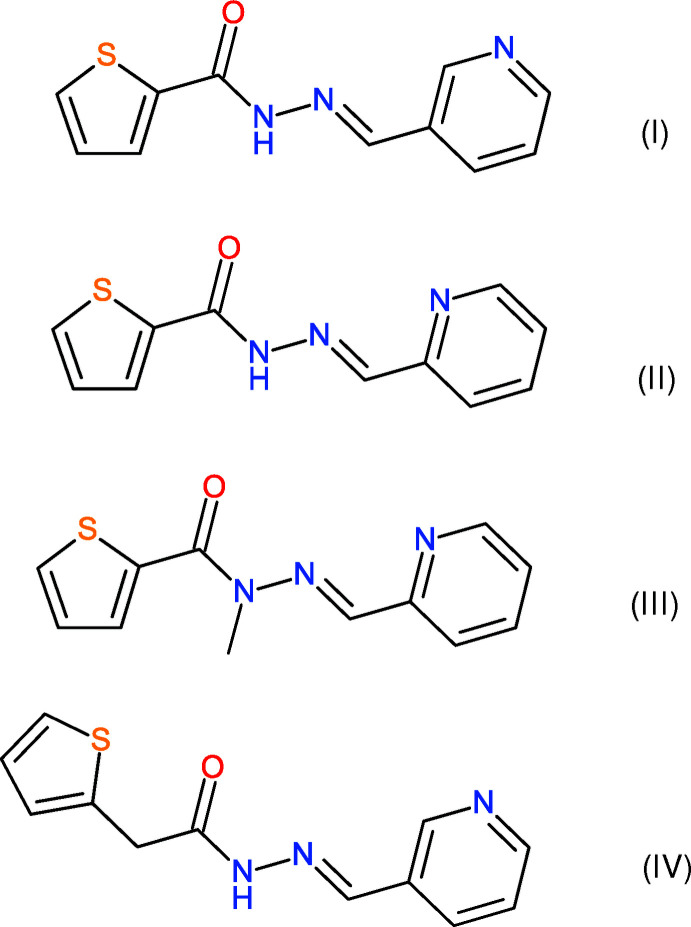


## 2. Structural commentary

**Figure 1:**
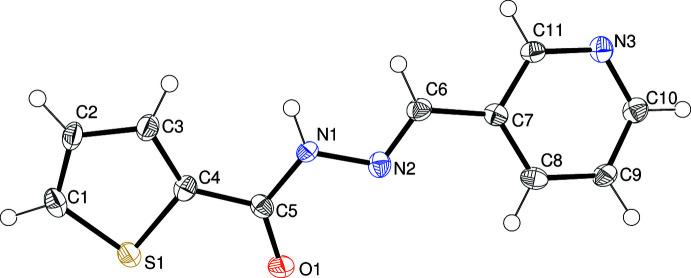
The mol­ecular structure of (I) showing 50% displacement ellipsoids.

The mol­ecular structures of (I)–(IV) are shown in Figs. 1 ▸–4 ▸ ▸ ▸, respectively and they all confirm the structures (atomic connectivities) postulated in the previous studies noted in the synthesis section: each compound crystallizes with one mol­ecule in the asymmetric unit and there is no suggestion that any of these compounds exist in the ‘enol’ —C(OH)=N— tautomer in the solid state.

In (I) (Fig. 1 ▸), the conformation about the N2=C6 bond [1.280 (5) Å] is *E* and the C5—N1—N2—C6 torsion angle is 175.1 (4)°. The oxygen atom of the carbonyl group and the sulfur atom of the thio­phene ring lie on the same side of the mol­ecule [S1—C4—C5—O1 = −4.9 (6)°] whereas atom N3 of the pyridine ring lies on the opposite side. The dihedral angle between the thio­phene and pyridine rings is 21.4 (2)° and the largest twist in the mol­ecule occurs about the C6—C7 bond [N2—C6—C7—C8 = −11.8 (7)°]. The N1—N2 bond length of 1.384 (5) Å in (I) is significantly shorter than a typical N—N single bond (∼1.44 Å), which suggests substantial delocalization of electrons with the adjacent C5=O1 carbonyl group and the N2=C6 double bond, as observed previously for related compounds (Cardoso *et al.*, 2016*c* ▸). Otherwise, the bond lengths and angles in (I) may be regarded as unexceptional.

**Figure 2:**
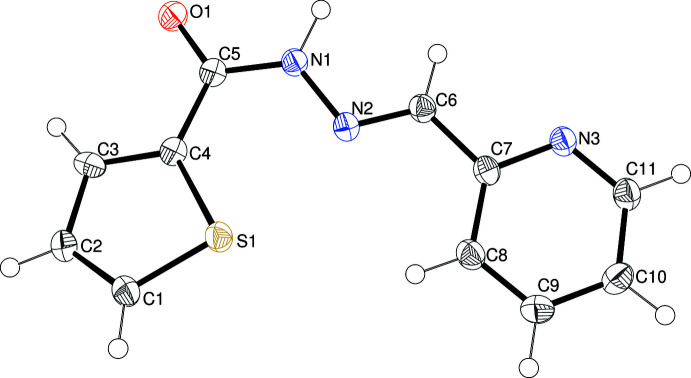
The mol­ecular structure of (II) showing 50% displacement ellipsoids.

In (II) (Fig. 2 ▸), the N2=C6 double bond [1.284 (3) Å] is also in an *E* configuration and C5—N1—N2—C6 = 173.74 (19)° but unlike (I), atoms O1 and S1 lie on opposite sides of the mol­ecule [S1—C4—C5—O1 = −170.67 (17)°] and N3 lies on the same side as O1. The dihedral angle between the aromatic rings is 15.42 (14)° and the most significant twist occurs about the C5—N1 bond [C4—C5—N1—N2 = 12.0 (3)°]. The C8—H8 bond of the pyridine ring points towards S1 but with H⋯S = 3.22 Å (sum of van der Waals radii = 3.00 Å) we consider it to be too long to be regarded as an intra­molecular hydrogen bond.

**Figure 3:**
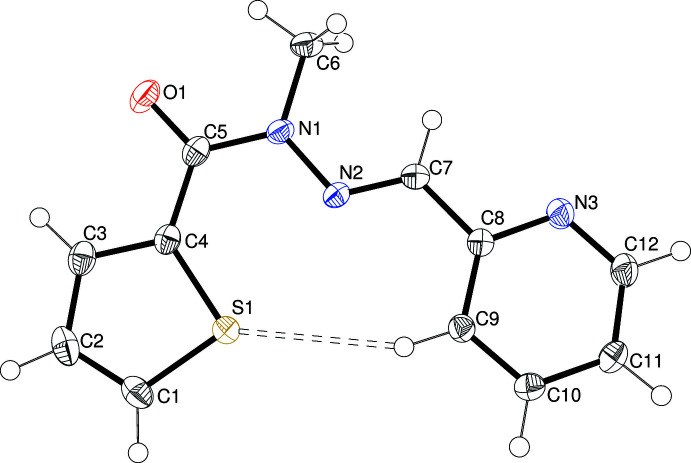
The mol­ecular structure of (III) showing 50% displacement ellipsoids. The short C—H⋯S contact is indicated by a double-dashed line.

Compound (III) (Fig. 3 ▸) is the *N*-methyl­ated derivative of (II): the N2=C7 bond [1.2815 (17) Å] has an *E* configuration and C5—N1—N2—C7 = 179.40 (12)°. As with (II), O1 and S1 lie on opposite sides of the mol­ecule [S1—C4—C5—O1 = 178.88 (10)° and N3 lies on the same side as O1. The dihedral angle between the C1–C4/S1 and C8–C12/N3 rings is 4.97 (8)°: most of this twist appear to be about the C7—C8 bond [N2—C7—C8—C9 = −4.8 (2)°] although the whole mol­ecule is close to flat [r.m.s. deviation for the 17 non-H atoms = 0.065 Å]. In this case, the short intra­molecular H⋯S contact between C9—H9 and S1 is 2.84 Å (C—H⋯S = 155°), considerably shorter than the equivalent contact in (II), and reasonable for this type of weak inter­action (Ghosh *et al.*, 2020 ▸).

**Figure 4:**
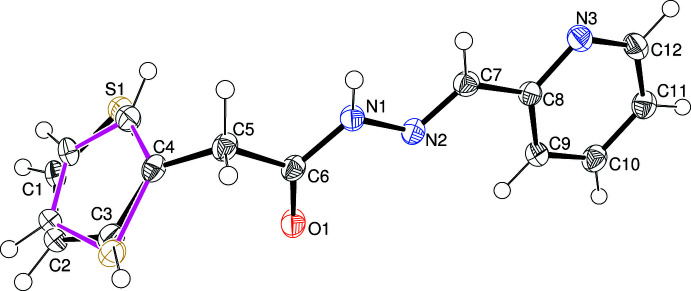
The mol­ecular structure of (IV) showing 50% displacement ellipsoids. The minor disorder component of the thio­phene ring is shown with pink bonds.

In (IV) (Fig. 4 ▸), the thio­phene ring was modelled with ‘flip’ disorder (∼180° rotation about the C4—C5 bond) in a 0.851 (2): 0.149 (2) ratio, which is a common structural feature for this moiety (Cardoso *et al.*, 2016*c* ▸). Once again, the configuration of the N2=C7 double bond [1.281 (2) Å] is *E* and C6 and C7 are close to *anti* about the N—N bond [C6—N1—N2—C7 = −177.90 (14)°]. The dihedral angle between the aromatic rings (major disorder conformation for the thio­phene moiety) in (IV) of 83.52 (13)° indicates near perpendicularity, which is quite different to the other compounds described here, presumably because the mol­ecule has additional conformational flexibility about the C—C single bonds associated with the C5 methyl­ene group [C3—C4—C5—C6 = 93.8 (6)°; C4—C5—C6—N1 = 144.72 (14)°].

## 3. Supra­molecular features

**Figure 5:**
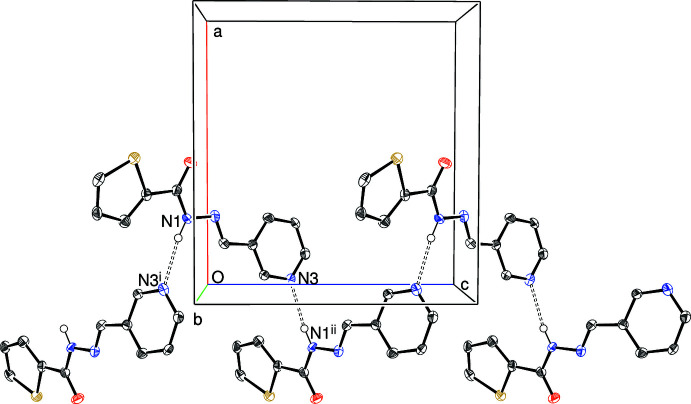
Fragment of the crystal structure of (I) showing part of an [001] *C*(7) chain linked by N—H⋯N hydrogen bonds (double dashed lines). Symmetry codes: (i) −*x*, −*y*, *z* − 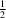 ; (ii) −*x*, −*y*, *z* + 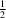 .

**Figure 6:**
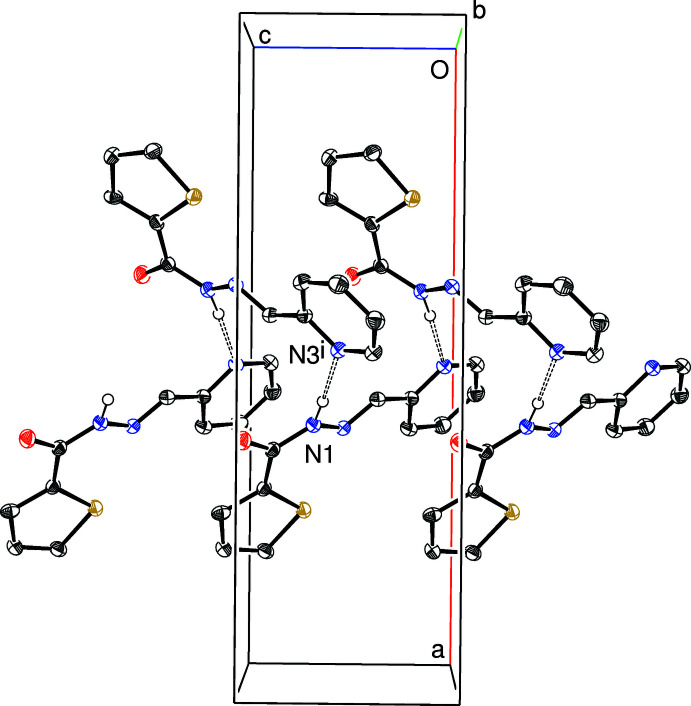
Fragment of the crystal structure of (II) showing part of an [001] *C*(6) chain linked by N—H⋯N hydrogen bonds (double-dashed lines). Symmetry code: (i) 1 − *x*, 1 − *y*, *z* + 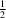 .

**Figure 7:**
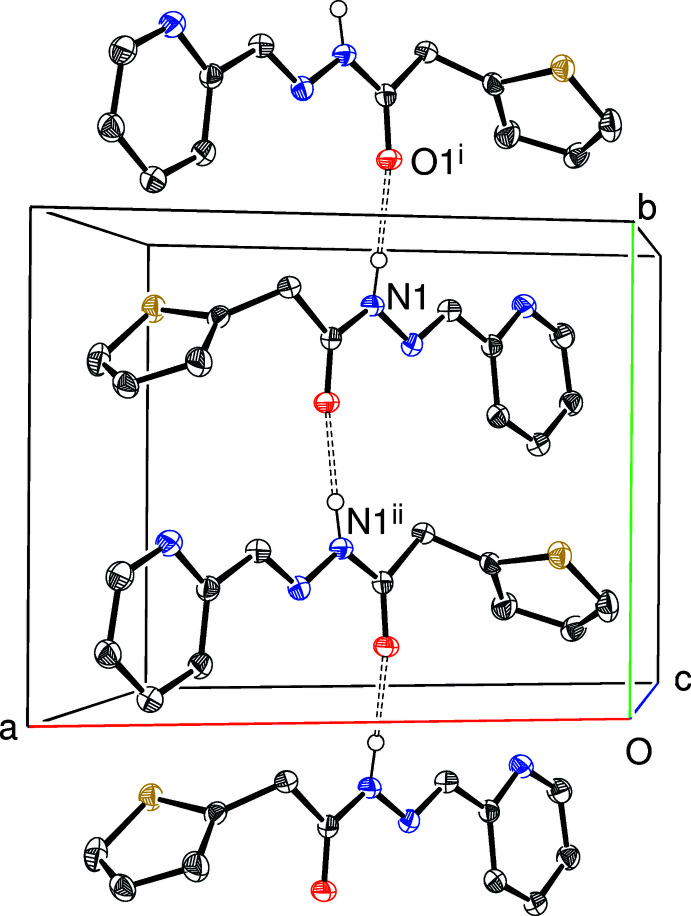
Fragment of the crystal structure of (IV) showing part of an [010] *C*(4) chain linked by N—H⋯O hydrogen bonds (double-dashed lines). Symmetry codes: (i) 1 − *x*, 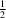 + *y*, 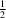 − *z*; (ii) 1 − *x*, *y* − 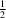 , 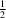 − *z*.

Geometrical data for the directional inter­molecular inter­actions in (I)–(IV) are listed in Tables 1 ▸–4 ▸ ▸ ▸, respectively. The most significant features in the packing of (I) and (II) are N—H⋯N_p_ (p = pyridine) hydrogen bonds: in the former, these links generate [001] *C*(7) chains (Fig. 5 ▸), with adjacent mol­ecules in the chain related by the 2_1_ screw axis. In (II), the equivalent inter­action also leads to [001] chains (Fig. 6 ▸) generated by the 2_1_ screw axis but here the graph-set motif is *C*(6). The packing for (IV) features classical *C*(4) amide N—H⋯O hydrogen bonds (Fig. 7 ▸) leading to [010] chains generated once again by a 2_1_ screw axis. There are obviously no classical hydrogen bonds in the extended structure of (III) and the only possible directional inter­molecular contact identified is a very weak C—H⋯N_p_ link arising from the *N*-methyl group. The structures of (I), (II) and (IV) also feature various C—H⋯*X* (*X* = N, O, S) inter­actions although these are presumably very weak, given their H⋯*X* lengths.

The shortest aromatic ring centroid–centroid separations in these structures are π_t_⋯π_p_ (t = thio­phene, p = pyridine) = 4.046 (2) Å (slippage = 1.546 Å) for (I), π_t_⋯π_p_ = 4.0509 (12) Å (slippage = 1.929 Å) for (II), π_t_⋯π_p_ = 4.7831 (9) Å for (III) and π_t_⋯π_p_ = 4.643 (2) Å for (IV). Given these distances, any aromatic ring-stacking effects that contribute to the cohesion and stability of the crystal must be weak to non-existent.

In order to gain more insight into these different packing motifs, the Hirshfeld surfaces and fingerprint plots for (I)–(IV) were calculated using *CrystalExplorer* (Turner *et al.*, 2017 ▸) following the approach recently described by Tan *et al.* (2019 ▸). The Hirshfeld surfaces (see supporting information) show the expected red spots (close contacts) in the vicinities of the various donor and acceptor atoms.

The fingerprint plots for (I)–(IV) decomposed into the different percentage contact types (Table 5 ▸) show that the different contributions are broadly similar, with H⋯H (van der Waals) contacts the most significant for each structure, followed by C⋯H/H⋯C. The O⋯H/H⋯O and N⋯H/H⋯N contributions are almost the same for the four structures, despite the lack of classical hydrogen bonds in (III). The S⋯H/H⋯S percentage contributions for (I) and (IV) are notably greater than those for (II) and (III), possibly because the S atom is ‘facing outwards’ in the former structures but is associated with an intra­molecular C—H⋯S close contact arising from the pyridine ring in the latter structures. It is notable that the percentage of O⋯O contacts is zero in all structures, presumably reflecting the fact that ‘bare’ O atoms avoid each other in the solid state for electrostatic reasons.

## 4. Database survey

A survey of the Cambridge Structural Database (CSD Core 2012.3 version of March 2022; Groom *et al.*, 2016 ▸) revealed nine structures incorporating the T—C(=O)—NH—N=CH—Q (T = thio­phene ring; Q = thio­phene or furan or pyrrole ring or derivatives) grouping and two with the T—CH_2_—C(=O)—NH—N=CH—Q sequence. None of these structures features a pyridine ring in the ‘Q’ position.

## 5. Synthesis and crystallization

Compounds (I) and (II) were prepared by a literature procedure (Lima *et al.*, 2000 ▸) and single crystals suitable for data collection were recrystallized from ethanol solution at room temperature. For the syntheses and spectroscopic characterizations of (III) and (IV), see Cardoso *et al.* (2016*a* ▸) and Cardoso *et al.* (2014 ▸), respectively: in each case, colourless blocks suitable for X-ray data collections were recrystallized from ethanol solution at room temperature.

## 6. Refinement

Crystal data, data collection and structure refinement details are summarized in Table 6 ▸. The thio­phene ring in (IV) was modelled as disordered over two sets of sites related by an approximate rotation of 180° about the C4—C5 bond in a 0.851 (2): 0.149 (2) ratio. EADP cards in *SHELXL* were used for the *U*_ij_ values of equivalent atom pairs (*e.g*., C1 and C1*B*) and a SAME card was used to restrain the nearest-neighbour and next-nearest-neighbour bond distances in the two disorder components to be equal with standard deviations of 0.02 and 0.04 Å, respectively. The N-bound H atoms in (I), (II) and (IV) were located in difference maps and their positions were freely refined with *U*_iso_(H) = 1.2*U*_eq_(N). All C-bound H atoms were located geometrically (C—H = 0.95–0.99 Å) and refined as riding atoms with *U*_iso_(H) = 1.2*U*_eq_(C) or 1.5*U*_eq_(methyl C). The methyl group in (III) was allowed to rotate, but not to tip, to best fit the electron density.

## Supplementary Material

Crystal structure: contains datablock(s) I, II, III, IV, global. DOI: 10.1107/S2056989022005151/zl5030sup1.cif

Structure factors: contains datablock(s) I. DOI: 10.1107/S2056989022005151/zl5030Isup2.hkl

Structure factors: contains datablock(s) II. DOI: 10.1107/S2056989022005151/zl5030IIsup3.hkl

Structure factors: contains datablock(s) III. DOI: 10.1107/S2056989022005151/zl5030IIIsup4.hkl

Structure factors: contains datablock(s) IV. DOI: 10.1107/S2056989022005151/zl5030IVsup5.hkl

Supporting information file. DOI: 10.1107/S2056989022005151/zl5030Isup6.cml

Supporting information file. DOI: 10.1107/S2056989022005151/zl5030IIsup7.cml

Supporting information file. DOI: 10.1107/S2056989022005151/zl5030IIIsup8.cml

Supporting information file. DOI: 10.1107/S2056989022005151/zl5030IVsup9.cml

Hirshfeld surfaces. DOI: 10.1107/S2056989022005151/zl5030sup10.docx

CCDC references: 2172437, 2172436, 2172435, 2172434

Additional supporting information:  crystallographic information; 3D view; checkCIF report

## Figures and Tables

**Table 1:** Hydrogen-bond geometry (Å, °) for (I)

*D*—H⋯*A*	*D*—H	H⋯*A*	*D*⋯*A*	*D*—H⋯*A*
N1—H1*N*⋯N3^i^	0.87 (5)	2.14 (5)	2.995 (5)	166 (4)
C1—H1⋯O1^ii^	0.95	2.53	3.471 (6)	171
C3—H3⋯N3^i^	0.95	2.61	3.479 (6)	152
C6—H6⋯N3^i^	0.95	2.59	3.410 (6)	145
C9—H9⋯O1^iii^	0.95	2.66	3.397 (5)	135
C11—H11⋯N2^iv^	0.95	2.57	3.481 (6)	160

Symmetry codes: (i) 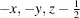 ; (ii) 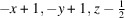 ; (iii) 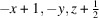 ; (iv) 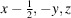 .

**Table 2:** Hydrogen-bond geometry (Å, °) for (II)

*D*—H⋯*A*	*D*—H	H⋯*A*	*D*⋯*A*	*D*—H⋯*A*
N1—H1*N*⋯N3^i^	0.98 (3)	2.03 (3)	3.013 (3)	177 (3)
C1—H1⋯O1^ii^	0.95	2.48	3.101 (3)	123
C2—H2⋯O1^iii^	0.95	2.64	3.410 (3)	139

Symmetry codes: (i) 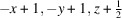 ; (ii) 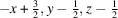 ; (iii) 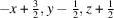 .

**Table 3:** Hydrogen-bond geometry (Å, °) for (III)

*D*—H⋯*A*	*D*—H	H⋯*A*	*D*⋯*A*	*D*—H⋯*A*
C9—H9⋯S1	0.95	2.84	3.7217 (13)	155
C6—H6*C*⋯N3^i^	0.98	2.61	3.3499 (18)	132

Symmetry code: (i) 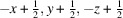 .

**Table 4:** Hydrogen-bond geometry (Å, °) for (IV)

*D*—H⋯*A*	*D*—H	H⋯*A*	*D*⋯*A*	*D*—H⋯*A*
N1—H1*N*⋯O1^i^	0.88 (2)	2.00 (2)	2.8628 (18)	164.9 (18)
C3—H3⋯N1^ii^	0.95	2.78	3.718 (7)	172
C5—H5*B*⋯O1^i^	0.99	2.64	3.307 (2)	125
C7—H7⋯S1*B* ^i^	0.95	2.65	3.534 (16)	155
C12—H12⋯S1^iii^	0.95	2.98	3.6624 (19)	129

Symmetry codes: (i) 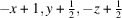 ; (ii) 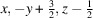 ; (iii) 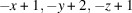 .

**Table 5:** Hirshfeld fingerprint contact percentages for (I)–(IV)

Contact type	(I)	(II)	(III)	(IV)^*a*^
H⋯H	30.1	32.8	36.5	34.5
C⋯H/H⋯C	15.1	23.3	28.2	22.6
O⋯H/H⋯O	13.1	12.8	10.4	11.2
N⋯H/H⋯N	13.7	12.2	11.5	13.8
S⋯H/H⋯S	12.1	7.0	5.8	10.7
C⋯C	6.2	4.5	1.8	1.2
C⋯O/O⋯C	1.3	0.8	0.7	1.0
O⋯O	0.0	0.0	0.0	0.0

Note: (*a*) Major disorder component.

**Table 6:** Experimental details

	(I)	(II)	(III)	(IV)
Crystal data				
Chemical formula	C_11_H_9_N_3_OS	C_11_H_9_N_3_OS	C_12_H_11_N_3_OS	C_12_H_11_N_3_OS
*M*_r_	231.27	231.27	245.30	245.30
Crystal system, space group	Orthorhombic, *P* *c* *a*2_1_	Orthorhombic, *P* *n* *a*2_1_	Monoclinic, *C*2/*c*	Monoclinic, *P*2_1_/*c*
Temperature (K)	100	100	100	100
*a*, *b*, *c* (Å)	10.6845 (9), 9.4974 (9), 10.0917 (10)	18.4056 (13), 9.5255 (7), 6.0300 (4)	21.0690 (15), 5.1085 (4), 21.1531 (15)	11.3963 (8), 9.2782 (7), 11.8178 (8)
α, β, γ (°)	90, 90, 90	90, 90, 90	90, 95.265 (2), 90	90, 112.761 (2), 90
*V* (Å^3^)	1024.05 (16)	1057.19 (13)	2267.1 (3)	1152.27 (14)
*Z*	4	4	8	4
Radiation type	Mo *K*α	Mo *K*α	Mo *K*α	Mo *K*α
μ (mm^−1^)	0.30	0.29	0.27	0.27
Crystal size (mm)	0.05 × 0.04 × 0.01	0.15 × 0.06 × 0.04	0.42 × 0.12 × 0.03	0.10 × 0.09 × 0.06

Data collection				
Diffractometer	Rigaku Saturn724+ CCD	Rigaku Saturn724+ CCD	Rigaku Saturn724+ CCD	Rigaku AFC12 CCD
Absorption correction	Multi-scan (*CrystalClear*; Rigaku, 2012 ▸)	Multi-scan (*CrystalClear*; Rigaku, 2012 ▸)	Multi-scan (*CrystalClear*; Rigaku, 2012 ▸)	Multi-scan (*CrystalClear*; Rigaku, 2012 ▸)
*T*_min_, *T*_max_	0.484, 1.000	0.756, 1.000	0.780, 1.000	0.723, 1.000
No. of measured, independent and observed [*I* > 2σ(*I*)] reflections	6740, 1822, 1559	7314, 1979, 1930	8717, 2563, 2307	8155, 2593, 2138
*R*_int_	0.058	0.026	0.022	0.031
(sin θ/λ)_max_ (Å^−1^)	0.649	0.649	0.650	0.670

Refinement				
*R*[*F* ^2^ > 2σ(*F* ^2^)], *wR*(*F* ^2^), *S*	0.046, 0.112, 1.08	0.029, 0.083, 1.08	0.031, 0.087, 1.07	0.041, 0.112, 1.10
No. of reflections	1822	1979	2563	2593
No. of parameters	148	148	155	170
No. of restraints	1	1	0	10
H-atom treatment	H atoms treated by a mixture of independent and constrained refinement	H atoms treated by a mixture of independent and constrained refinement	H-atom parameters constrained	H atoms treated by a mixture of independent and constrained refinement
Δρ_max_, Δρ_min_ (e Å^−3^)	0.27, −0.43	0.33, −0.24	0.33, −0.29	0.32, −0.30
Absolute structure	Parsons *et al.* (2013 ▸)	Parsons *et al.* (2013 ▸)	–	–
Absolute structure parameter	0.02 (13)	0.04 (4)	–	–

Computer programs: *CrystalClear* (Rigaku, 2012 ▸), *SHELXS97* (Sheldrick, 2008 ▸), *SHELXL2018/3* (Sheldrick, 2015 ▸), *ORTEP-3 for Windows* (Farrugia, 2012 ▸) and *publCIF* (Westrip, 2010 ▸).

## supplementary crystallographic information

### N'-[(E)-Pyridin-3-ylmethylidene]thiophene-2-carbohydrazide (I) Crystal data

**Table:**

C_11_H_9_N_3_OS	*D*_x_ = 1.500 Mg m^−^^3^
*M**_r_* = 231.27	Mo *K*α radiation, λ = 0.71073 Å
Orthorhombic, *P**c**a*2_1_	Cell parameters from 5861 reflections
*a* = 10.6845 (9) Å	θ = 3.5–27.5°
*b* = 9.4974 (9) Å	µ = 0.30 mm^−^^1^
*c* = 10.0917 (10) Å	*T* = 100 K
*V* = 1024.05 (16) Å^3^	Chip, colourless
*Z* = 4	0.05 × 0.04 × 0.01 mm
*F*(000) = 480	

### N'-[(E)-Pyridin-3-ylmethylidene]thiophene-2-carbohydrazide (I) Data collection

**Table:**

Rigaku Saturn724+ CCD diffractometer	1559 reflections with *I* > 2σ(*I*)
ω scans	*R*_int_ = 0.058
Absorption correction: multi-scan (CrystalClear; Rigaku, 2012)	θ_max_ = 27.5°, θ_min_ = 3.5°
*T*_min_ = 0.484, *T*_max_ = 1.000	*h* = −12→13
6740 measured reflections	*k* = −12→12
1822 independent reflections	*l* = −7→13

### N'-[(E)-Pyridin-3-ylmethylidene]thiophene-2-carbohydrazide (I) Refinement

**Table:**

Refinement on *F*^2^	Hydrogen site location: mixed
Least-squares matrix: full	H atoms treated by a mixture of independent and constrained refinement
*R*[*F*^2^ > 2σ(*F*^2^)] = 0.046	*w* = 1/[σ^2^(*F*_o_^2^) + (0.0492*P*)^2^ + 0.5837*P*] where *P* = (*F*_o_^2^ + 2*F*_c_^2^)/3
*wR*(*F*^2^) = 0.112	(Δ/σ)_max_ < 0.001
*S* = 1.08	Δρ_max_ = 0.27 e Å^−^^3^
1822 reflections	Δρ_min_ = −0.43 e Å^−^^3^
148 parameters	Absolute structure: Parsons *et al.* (2013)
1 restraint	Absolute structure parameter: 0.02 (13)
Primary atom site location: structure-invariant direct methods	

### N'-[(E)-Pyridin-3-ylmethylidene]thiophene-2-carbohydrazide (I) Special details

**Table:**

Geometry. All esds (except the esd in the dihedral angle between two l.s. planes) are estimated using the full covariance matrix. The cell esds are taken into account individually in the estimation of esds in distances, angles and torsion angles; correlations between esds in cell parameters are only used when they are defined by crystal symmetry. An approximate (isotropic) treatment of cell esds is used for estimating esds involving l.s. planes.

### N'-[(E)-Pyridin-3-ylmethylidene]thiophene-2-carbohydrazide (I) Fractional atomic coordinates and isotropic or equivalent isotropic displacement parameters (Å^2^)

**Table:**

	*x*	*y*	*z*	*U*_iso_*/*U*_eq_	
C1	0.4074 (4)	0.5100 (5)	−0.3833 (5)	0.0253 (10)	
H1	0.444915	0.579791	−0.437808	0.030*	
C2	0.2866 (4)	0.4653 (4)	−0.3959 (5)	0.0239 (10)	
H2	0.230294	0.500393	−0.460776	0.029*	
C3	0.2544 (4)	0.3608 (4)	−0.3018 (5)	0.0236 (10)	
H3	0.173924	0.318696	−0.296656	0.028*	
C4	0.3510 (4)	0.3271 (4)	−0.2195 (4)	0.0199 (10)	
C5	0.3644 (4)	0.2257 (4)	−0.1092 (4)	0.0194 (9)	
C6	0.1581 (4)	0.0118 (5)	0.0671 (5)	0.0215 (9)	
H6	0.084911	0.035530	0.018411	0.026*	
C7	0.1483 (4)	−0.0844 (4)	0.1798 (4)	0.0188 (9)	
C8	0.2501 (3)	−0.1470 (4)	0.2449 (6)	0.0215 (9)	
H8	0.333133	−0.132248	0.214145	0.026*	
C9	0.2277 (4)	−0.2306 (4)	0.3544 (5)	0.0208 (9)	
H9	0.295199	−0.273712	0.400284	0.025*	
C10	0.1052 (4)	−0.2507 (5)	0.3966 (5)	0.0228 (10)	
H10	0.091113	−0.306383	0.473419	0.027*	
C11	0.0286 (3)	−0.1154 (4)	0.2275 (5)	0.0196 (9)	
H11	−0.041192	−0.077506	0.181272	0.023*	
N1	0.2581 (3)	0.1569 (4)	−0.0731 (4)	0.0196 (8)	
H1N	0.186 (4)	0.181 (5)	−0.107 (5)	0.023*	
N2	0.2634 (3)	0.0646 (4)	0.0328 (4)	0.0211 (8)	
N3	0.0061 (3)	−0.1955 (4)	0.3344 (4)	0.0217 (8)	
O1	0.4666 (3)	0.2073 (3)	−0.0557 (4)	0.0278 (8)	
S1	0.48271 (9)	0.42487 (11)	−0.25732 (14)	0.0248 (3)	

### N'-[(E)-Pyridin-3-ylmethylidene]thiophene-2-carbohydrazide (I) Atomic displacement parameters (Å^2^)

**Table:**

	*U*^11^	*U*^22^	*U*^33^	*U*^12^	*U*^13^	*U*^23^
C1	0.030 (2)	0.024 (2)	0.022 (3)	−0.0009 (18)	0.009 (2)	0.002 (2)
C2	0.030 (2)	0.026 (2)	0.016 (3)	0.0038 (18)	0.000 (2)	0.006 (2)
C3	0.026 (2)	0.022 (2)	0.022 (3)	0.0033 (16)	−0.0001 (18)	0.0045 (19)
C4	0.025 (2)	0.0204 (19)	0.015 (2)	0.0014 (16)	−0.0012 (18)	−0.0021 (17)
C5	0.020 (2)	0.022 (2)	0.017 (2)	−0.0029 (16)	0.0016 (18)	−0.0019 (19)
C6	0.0196 (19)	0.025 (2)	0.019 (2)	−0.0002 (17)	−0.004 (2)	−0.0007 (19)
C7	0.018 (2)	0.0216 (19)	0.017 (2)	−0.0014 (15)	0.0001 (18)	−0.0027 (18)
C8	0.0177 (18)	0.0242 (19)	0.023 (2)	−0.0026 (13)	−0.002 (3)	−0.005 (2)
C9	0.020 (2)	0.026 (2)	0.017 (2)	0.0002 (16)	−0.006 (2)	0.001 (2)
C10	0.025 (2)	0.027 (2)	0.017 (2)	0.0016 (17)	−0.001 (2)	−0.0001 (19)
C11	0.0169 (17)	0.0238 (18)	0.018 (2)	0.0012 (14)	−0.001 (2)	−0.002 (2)
N1	0.0164 (17)	0.0258 (18)	0.016 (2)	−0.0001 (14)	−0.0040 (16)	0.0038 (16)
N2	0.0247 (19)	0.0223 (18)	0.016 (2)	−0.0014 (14)	−0.0010 (18)	0.0000 (16)
N3	0.0250 (18)	0.0222 (16)	0.018 (2)	0.0005 (15)	0.0031 (17)	−0.0009 (16)
O1	0.0179 (15)	0.0385 (18)	0.0270 (19)	−0.0039 (12)	−0.0050 (15)	0.0063 (17)
S1	0.0222 (5)	0.0301 (5)	0.0221 (6)	−0.0036 (4)	0.0015 (6)	0.0037 (6)

### N'-[(E)-Pyridin-3-ylmethylidene]thiophene-2-carbohydrazide (I) Geometric parameters (Å, º)

**Table:**

C1—C2	1.364 (6)	C6—H6	0.9500
C1—S1	1.708 (5)	C7—C11	1.398 (5)
C1—H1	0.9500	C7—C8	1.403 (6)
C2—C3	1.415 (6)	C8—C9	1.381 (7)
C2—H2	0.9500	C8—H8	0.9500
C3—C4	1.363 (6)	C9—C10	1.390 (6)
C3—H3	0.9500	C9—H9	0.9500
C4—C5	1.479 (6)	C10—N3	1.338 (5)
C4—S1	1.729 (4)	C10—H10	0.9500
C5—O1	1.230 (5)	C11—N3	1.342 (6)
C5—N1	1.360 (5)	C11—H11	0.9500
C6—N2	1.280 (5)	N1—N2	1.384 (5)
C6—C7	1.463 (6)	N1—H1N	0.87 (5)
			
C2—C1—S1	111.6 (3)	C8—C7—C6	125.0 (4)
C2—C1—H1	124.2	C9—C8—C7	118.9 (4)
S1—C1—H1	124.2	C9—C8—H8	120.5
C1—C2—C3	112.7 (4)	C7—C8—H8	120.5
C1—C2—H2	123.7	C8—C9—C10	119.2 (4)
C3—C2—H2	123.7	C8—C9—H9	120.4
C4—C3—C2	112.9 (4)	C10—C9—H9	120.4
C4—C3—H3	123.5	N3—C10—C9	123.2 (4)
C2—C3—H3	123.5	N3—C10—H10	118.4
C3—C4—C5	133.3 (4)	C9—C10—H10	118.4
C3—C4—S1	110.8 (3)	N3—C11—C7	124.1 (4)
C5—C4—S1	115.9 (3)	N3—C11—H11	118.0
O1—C5—N1	123.7 (4)	C7—C11—H11	118.0
O1—C5—C4	120.6 (4)	C5—N1—N2	118.5 (4)
N1—C5—C4	115.7 (4)	C5—N1—H1N	120 (3)
N2—C6—C7	121.1 (4)	N2—N1—H1N	120 (3)
N2—C6—H6	119.4	C6—N2—N1	114.8 (4)
C7—C6—H6	119.4	C10—N3—C11	117.3 (4)
C11—C7—C8	117.3 (4)	C1—S1—C4	92.0 (2)
C11—C7—C6	117.7 (4)		
			
S1—C1—C2—C3	0.2 (5)	C8—C9—C10—N3	1.6 (7)
C1—C2—C3—C4	−0.3 (6)	C8—C7—C11—N3	3.2 (6)
C2—C3—C4—C5	−179.1 (5)	C6—C7—C11—N3	−176.2 (4)
C2—C3—C4—S1	0.2 (5)	O1—C5—N1—N2	2.4 (6)
C3—C4—C5—O1	174.4 (5)	C4—C5—N1—N2	−177.6 (3)
S1—C4—C5—O1	−4.9 (6)	C7—C6—N2—N1	−178.2 (4)
C3—C4—C5—N1	−5.6 (7)	C5—N1—N2—C6	175.1 (4)
S1—C4—C5—N1	175.1 (3)	C9—C10—N3—C11	−1.0 (6)
N2—C6—C7—C11	167.6 (4)	C7—C11—N3—C10	−1.5 (6)
N2—C6—C7—C8	−11.8 (7)	C2—C1—S1—C4	−0.1 (4)
C11—C7—C8—C9	−2.5 (6)	C3—C4—S1—C1	−0.1 (4)
C6—C7—C8—C9	176.9 (4)	C5—C4—S1—C1	179.4 (3)
C7—C8—C9—C10	0.3 (6)		

### N'-[(E)-Pyridin-3-ylmethylidene]thiophene-2-carbohydrazide (I) Hydrogen-bond geometry (Å, º)

**Table:**

*D*—H···*A*	*D*—H	H···*A*	*D*···*A*	*D*—H···*A*
N1—H1*N*···N3^i^	0.87 (5)	2.14 (5)	2.995 (5)	166 (4)
C1—H1···O1^ii^	0.95	2.53	3.471 (6)	171
C3—H3···N3^i^	0.95	2.61	3.479 (6)	152
C6—H6···N3^i^	0.95	2.59	3.410 (6)	145
C9—H9···O1^iii^	0.95	2.66	3.397 (5)	135
C11—H11···N2^iv^	0.95	2.57	3.481 (6)	160

Symmetry codes: (i) −*x*, −*y*, *z*−1/2; (ii) −*x*+1, −*y*+1, *z*−1/2; (iii) −*x*+1, −*y*, *z*+1/2; (iv) *x*−1/2, −*y*, *z*.

### N'-[(E)-Pyridin-2-ylmethylidene]thiophene-2-carbohydrazide (II) Crystal data

**Table:**

C_11_H_9_N_3_OS	*D*_x_ = 1.453 Mg m^−^^3^
*M**_r_* = 231.27	Mo *K*α radiation, λ = 0.71073 Å
Orthorhombic, *P**n**a*2_1_	Cell parameters from 6000 reflections
*a* = 18.4056 (13) Å	θ = 2.4–27.5°
*b* = 9.5255 (7) Å	µ = 0.29 mm^−^^1^
*c* = 6.0300 (4) Å	*T* = 100 K
*V* = 1057.19 (13) Å^3^	Blade, dark orange
*Z* = 4	0.15 × 0.06 × 0.04 mm
*F*(000) = 480	

### N'-[(E)-Pyridin-2-ylmethylidene]thiophene-2-carbohydrazide (II) Data collection

**Table:**

Rigaku Saturn724+ CCD diffractometer	1930 reflections with *I* > 2σ(*I*)
ω scans	*R*_int_ = 0.026
Absorption correction: multi-scan (CrystalClear; Rigaku, 2012)	θ_max_ = 27.5°, θ_min_ = 3.1°
*T*_min_ = 0.756, *T*_max_ = 1.000	*h* = −23→23
7314 measured reflections	*k* = −9→12
1979 independent reflections	*l* = −5→7

### N'-[(E)-Pyridin-2-ylmethylidene]thiophene-2-carbohydrazide (II) Refinement

**Table:**

Refinement on *F*^2^	Hydrogen site location: mixed
Least-squares matrix: full	H atoms treated by a mixture of independent and constrained refinement
*R*[*F*^2^ > 2σ(*F*^2^)] = 0.029	*w* = 1/[σ^2^(*F*_o_^2^) + (0.0573*P*)^2^ + 0.2224*P*] where *P* = (*F*_o_^2^ + 2*F*_c_^2^)/3
*wR*(*F*^2^) = 0.083	(Δ/σ)_max_ = 0.001
*S* = 1.08	Δρ_max_ = 0.33 e Å^−^^3^
1979 reflections	Δρ_min_ = −0.24 e Å^−^^3^
148 parameters	Absolute structure: Parsons *et al.* (2013)
1 restraint	Absolute structure parameter: 0.04 (4)
Primary atom site location: structure-invariant direct methods	

### N'-[(E)-Pyridin-2-ylmethylidene]thiophene-2-carbohydrazide (II) Special details

**Table:**

Geometry. All esds (except the esd in the dihedral angle between two l.s. planes) are estimated using the full covariance matrix. The cell esds are taken into account individually in the estimation of esds in distances, angles and torsion angles; correlations between esds in cell parameters are only used when they are defined by crystal symmetry. An approximate (isotropic) treatment of cell esds is used for estimating esds involving l.s. planes.

### N'-[(E)-Pyridin-2-ylmethylidene]thiophene-2-carbohydrazide (II) Fractional atomic coordinates and isotropic or equivalent isotropic displacement parameters (Å^2^)

**Table:**

	*x*	*y*	*z*	*U*_iso_*/*U*_eq_	
C1	0.80728 (12)	0.2567 (2)	0.9079 (4)	0.0243 (5)	
H1	0.844507	0.190518	0.876806	0.029*	
C2	0.80182 (12)	0.3272 (2)	1.1043 (4)	0.0240 (5)	
H2	0.834112	0.312980	1.225101	0.029*	
C3	0.74255 (10)	0.4248 (2)	1.1102 (5)	0.0225 (5)	
H3	0.730933	0.483815	1.232138	0.027*	
C4	0.70395 (11)	0.4200 (2)	0.9075 (4)	0.0198 (4)	
C5	0.64198 (11)	0.5159 (2)	0.8639 (4)	0.0201 (4)	
C6	0.56443 (11)	0.3875 (2)	0.3737 (4)	0.0214 (4)	
H6	0.530355	0.461814	0.358627	0.026*	
C7	0.56402 (10)	0.2730 (2)	0.2119 (5)	0.0207 (4)	
C8	0.61299 (11)	0.1601 (2)	0.2192 (5)	0.0248 (4)	
H8	0.647838	0.152750	0.334814	0.030*	
C9	0.60922 (12)	0.0599 (2)	0.0540 (5)	0.0282 (5)	
H9	0.642008	−0.017145	0.053144	0.034*	
C10	0.55693 (12)	0.0730 (2)	−0.1110 (5)	0.0276 (5)	
H10	0.553393	0.005274	−0.226127	0.033*	
C11	0.51001 (12)	0.1869 (2)	−0.1042 (5)	0.0252 (5)	
H11	0.474376	0.195559	−0.217400	0.030*	
N1	0.60295 (9)	0.50362 (19)	0.6733 (3)	0.0207 (4)	
H1N	0.5663 (14)	0.573 (3)	0.629 (5)	0.025*	
N2	0.60975 (9)	0.38992 (19)	0.5354 (3)	0.0200 (4)	
N3	0.51261 (10)	0.2853 (2)	0.0546 (4)	0.0222 (4)	
O1	0.62639 (9)	0.60967 (18)	0.9963 (3)	0.0263 (4)	
S1	0.74058 (3)	0.29964 (5)	0.72453 (13)	0.02379 (16)	

### N'-[(E)-Pyridin-2-ylmethylidene]thiophene-2-carbohydrazide (II) Atomic displacement parameters (Å^2^)

**Table:**

	*U*^11^	*U*^22^	*U*^33^	*U*^12^	*U*^13^	*U*^23^
C1	0.0204 (9)	0.0272 (11)	0.0252 (13)	0.0009 (8)	−0.0035 (9)	−0.0004 (11)
C2	0.0236 (10)	0.0273 (10)	0.0209 (12)	−0.0006 (9)	−0.0040 (9)	−0.0004 (10)
C3	0.0180 (9)	0.0194 (9)	0.0302 (14)	0.0013 (7)	0.0020 (9)	0.0050 (10)
C4	0.0203 (8)	0.0214 (9)	0.0177 (11)	−0.0019 (7)	0.0025 (8)	0.0003 (9)
C5	0.0201 (9)	0.0217 (9)	0.0184 (11)	−0.0016 (7)	0.0022 (8)	0.0005 (9)
C6	0.0180 (8)	0.0250 (10)	0.0213 (11)	0.0017 (7)	−0.0001 (9)	−0.0007 (9)
C7	0.0177 (8)	0.0244 (10)	0.0200 (11)	−0.0014 (7)	−0.0005 (10)	0.0021 (11)
C8	0.0205 (9)	0.0279 (10)	0.0258 (12)	0.0017 (7)	−0.0027 (10)	0.0002 (12)
C9	0.0268 (10)	0.0272 (11)	0.0307 (14)	0.0061 (9)	0.0010 (10)	−0.0019 (11)
C10	0.0275 (10)	0.0281 (11)	0.0271 (14)	−0.0002 (9)	0.0022 (10)	−0.0066 (10)
C11	0.0221 (9)	0.0306 (11)	0.0229 (13)	0.0000 (8)	−0.0032 (10)	−0.0009 (10)
N1	0.0199 (8)	0.0231 (8)	0.0189 (11)	0.0017 (7)	0.0003 (7)	−0.0012 (8)
N2	0.0181 (7)	0.0239 (8)	0.0178 (9)	−0.0005 (6)	0.0010 (7)	0.0006 (8)
N3	0.0202 (8)	0.0262 (9)	0.0203 (11)	0.0011 (6)	−0.0014 (8)	−0.0003 (8)
O1	0.0283 (8)	0.0284 (8)	0.0222 (10)	0.0038 (6)	−0.0002 (7)	−0.0046 (7)
S1	0.0226 (2)	0.0288 (3)	0.0200 (3)	0.00348 (18)	−0.0016 (3)	−0.0026 (3)

### N'-[(E)-Pyridin-2-ylmethylidene]thiophene-2-carbohydrazide (II) Geometric parameters (Å, º)

**Table:**

C1—C2	1.365 (4)	C6—H6	0.9500
C1—S1	1.702 (2)	C7—N3	1.345 (3)
C1—H1	0.9500	C7—C8	1.404 (3)
C2—C3	1.434 (3)	C8—C9	1.381 (4)
C2—H2	0.9500	C8—H8	0.9500
C3—C4	1.415 (3)	C9—C10	1.390 (4)
C3—H3	0.9500	C9—H9	0.9500
C4—C5	1.485 (3)	C10—C11	1.387 (3)
C4—S1	1.728 (2)	C10—H10	0.9500
C5—O1	1.232 (3)	C11—N3	1.341 (3)
C5—N1	1.361 (3)	C11—H11	0.9500
C6—N2	1.284 (3)	N1—N2	1.371 (3)
C6—C7	1.463 (3)	N1—H1N	0.98 (3)
			
C2—C1—S1	113.11 (17)	C8—C7—C6	123.1 (2)
C2—C1—H1	123.4	C9—C8—C7	118.3 (2)
S1—C1—H1	123.4	C9—C8—H8	120.8
C1—C2—C3	113.4 (2)	C7—C8—H8	120.8
C1—C2—H2	123.3	C8—C9—C10	119.3 (2)
C3—C2—H2	123.3	C8—C9—H9	120.4
C4—C3—C2	109.9 (2)	C10—C9—H9	120.4
C4—C3—H3	125.1	C11—C10—C9	118.7 (2)
C2—C3—H3	125.1	C11—C10—H10	120.6
C3—C4—C5	121.3 (2)	C9—C10—H10	120.6
C3—C4—S1	112.16 (16)	N3—C11—C10	123.0 (2)
C5—C4—S1	126.50 (18)	N3—C11—H11	118.5
O1—C5—N1	119.15 (19)	C10—C11—H11	118.5
O1—C5—C4	120.7 (2)	C5—N1—N2	122.15 (18)
N1—C5—C4	120.1 (2)	C5—N1—H1N	122.2 (17)
N2—C6—C7	121.55 (19)	N2—N1—H1N	115.5 (17)
N2—C6—H6	119.2	C6—N2—N1	114.55 (19)
C7—C6—H6	119.2	C11—N3—C7	117.90 (19)
N3—C7—C8	122.7 (2)	C1—S1—C4	91.48 (12)
N3—C7—C6	114.14 (18)		
			
S1—C1—C2—C3	−1.8 (3)	C8—C9—C10—C11	0.0 (4)
C1—C2—C3—C4	1.0 (3)	C9—C10—C11—N3	−0.1 (4)
C2—C3—C4—C5	−176.80 (19)	O1—C5—N1—N2	−169.81 (19)
C2—C3—C4—S1	0.2 (2)	C4—C5—N1—N2	12.0 (3)
C3—C4—C5—O1	5.8 (3)	C7—C6—N2—N1	−179.61 (19)
S1—C4—C5—O1	−170.67 (17)	C5—N1—N2—C6	173.74 (19)
C3—C4—C5—N1	−176.02 (19)	C10—C11—N3—C7	1.0 (3)
S1—C4—C5—N1	7.5 (3)	C8—C7—N3—C11	−1.9 (3)
N2—C6—C7—N3	179.2 (2)	C6—C7—N3—C11	178.0 (2)
N2—C6—C7—C8	−1.0 (4)	C2—C1—S1—C4	1.60 (19)
N3—C7—C8—C9	1.8 (4)	C3—C4—S1—C1	−0.99 (17)
C6—C7—C8—C9	−178.0 (2)	C5—C4—S1—C1	175.80 (19)
C7—C8—C9—C10	−0.8 (4)		

### N'-[(E)-Pyridin-2-ylmethylidene]thiophene-2-carbohydrazide (II) Hydrogen-bond geometry (Å, º)

**Table:**

*D*—H···*A*	*D*—H	H···*A*	*D*···*A*	*D*—H···*A*
N1—H1*N*···N3^i^	0.98 (3)	2.03 (3)	3.013 (3)	177 (3)
C1—H1···O1^ii^	0.95	2.48	3.101 (3)	123
C2—H2···O1^iii^	0.95	2.64	3.410 (3)	139

Symmetry codes: (i) −*x*+1, −*y*+1, *z*+1/2; (ii) −*x*+3/2, *y*−1/2, *z*−1/2; (iii) −*x*+3/2, *y*−1/2, *z*+1/2.

### N-Methyl-N'-[(E)-pyridin-2-ylmethylidene]thiophene-2-carbohydrazide (III) Crystal data

**Table:**

C_12_H_11_N_3_OS	*F*(000) = 1024
*M**_r_* = 245.30	*D*_x_ = 1.437 Mg m^−^^3^
Monoclinic, *C*2/*c*	Mo *K*α radiation, λ = 0.71073 Å
*a* = 21.0690 (15) Å	Cell parameters from 7400 reflections
*b* = 5.1085 (4) Å	θ = 2.9–27.5°
*c* = 21.1531 (15) Å	µ = 0.27 mm^−^^1^
β = 95.265 (2)°	*T* = 100 K
*V* = 2267.1 (3) Å^3^	Lath, colourless
*Z* = 8	0.42 × 0.12 × 0.03 mm

### N-Methyl-N'-[(E)-pyridin-2-ylmethylidene]thiophene-2-carbohydrazide (III) Data collection

**Table:**

Rigaku Saturn724+ CCD diffractometer	2307 reflections with *I* > 2σ(*I*)
ω scans	*R*_int_ = 0.022
Absorption correction: multi-scan (CrystalClear; Rigaku, 2012)	θ_max_ = 27.5°, θ_min_ = 2.9°
*T*_min_ = 0.780, *T*_max_ = 1.000	*h* = −27→27
8717 measured reflections	*k* = −6→6
2563 independent reflections	*l* = −27→21

### N-Methyl-N'-[(E)-pyridin-2-ylmethylidene]thiophene-2-carbohydrazide (III) Refinement

**Table:**

Refinement on *F*^2^	Primary atom site location: structure-invariant direct methods
Least-squares matrix: full	Hydrogen site location: inferred from neighbouring sites
*R*[*F*^2^ > 2σ(*F*^2^)] = 0.031	H-atom parameters constrained
*wR*(*F*^2^) = 0.087	*w* = 1/[σ^2^(*F*_o_^2^) + (0.0408*P*)^2^ + 2.1991*P*] where *P* = (*F*_o_^2^ + 2*F*_c_^2^)/3
*S* = 1.07	(Δ/σ)_max_ = 0.001
2563 reflections	Δρ_max_ = 0.33 e Å^−^^3^
155 parameters	Δρ_min_ = −0.29 e Å^−^^3^
0 restraints	

### N-Methyl-N'-[(E)-pyridin-2-ylmethylidene]thiophene-2-carbohydrazide (III) Special details

**Table:**

Geometry. All esds (except the esd in the dihedral angle between two l.s. planes) are estimated using the full covariance matrix. The cell esds are taken into account individually in the estimation of esds in distances, angles and torsion angles; correlations between esds in cell parameters are only used when they are defined by crystal symmetry. An approximate (isotropic) treatment of cell esds is used for estimating esds involving l.s. planes.

### N-Methyl-N'-[(E)-pyridin-2-ylmethylidene]thiophene-2-carbohydrazide (III) Fractional atomic coordinates and isotropic or equivalent isotropic displacement parameters (Å^2^)

**Table:**

	*x*	*y*	*z*	*U*_iso_*/*U*_eq_	
C1	0.50199 (7)	0.8774 (3)	0.34463 (7)	0.0262 (3)	
H1	0.525890	0.988871	0.319795	0.031*	
C2	0.50475 (7)	0.8871 (3)	0.40904 (7)	0.0283 (3)	
H2	0.530839	1.006088	0.434266	0.034*	
C3	0.46445 (6)	0.7003 (3)	0.43432 (6)	0.0243 (3)	
H3	0.460459	0.680014	0.478420	0.029*	
C4	0.43135 (6)	0.5499 (3)	0.38752 (6)	0.0196 (3)	
C5	0.38600 (6)	0.3464 (3)	0.40503 (6)	0.0206 (3)	
C6	0.30541 (6)	0.0098 (3)	0.37672 (6)	0.0229 (3)	
H6A	0.306676	0.001379	0.423104	0.034*	
H6B	0.316415	−0.161776	0.360136	0.034*	
H6C	0.262463	0.059115	0.359010	0.034*	
C7	0.32615 (6)	0.1249 (3)	0.25363 (6)	0.0190 (3)	
H7	0.298098	−0.007769	0.265792	0.023*	
C8	0.33189 (6)	0.1729 (2)	0.18583 (6)	0.0178 (3)	
C9	0.36845 (6)	0.3762 (3)	0.16384 (6)	0.0197 (3)	
H9	0.392055	0.489970	0.192720	0.024*	
C10	0.36941 (6)	0.4078 (3)	0.09876 (6)	0.0222 (3)	
H10	0.393440	0.545017	0.082220	0.027*	
C11	0.33481 (7)	0.2364 (3)	0.05837 (6)	0.0247 (3)	
H11	0.334717	0.253470	0.013642	0.030*	
C12	0.30036 (7)	0.0398 (3)	0.08438 (6)	0.0266 (3)	
H12	0.277110	−0.078189	0.056315	0.032*	
N1	0.35096 (5)	0.2040 (2)	0.35878 (5)	0.0194 (2)	
N2	0.35836 (5)	0.2591 (2)	0.29655 (5)	0.0176 (2)	
N3	0.29791 (6)	0.0063 (2)	0.14694 (5)	0.0234 (3)	
O1	0.37945 (5)	0.3068 (2)	0.46105 (4)	0.0291 (2)	
S1	0.45075 (2)	0.64215 (7)	0.31337 (2)	0.02308 (11)	

### N-Methyl-N'-[(E)-pyridin-2-ylmethylidene]thiophene-2-carbohydrazide (III) Atomic displacement parameters (Å^2^)

**Table:**

	*U*^11^	*U*^22^	*U*^33^	*U*^12^	*U*^13^	*U*^23^
C1	0.0201 (7)	0.0236 (7)	0.0350 (8)	−0.0028 (6)	0.0027 (5)	−0.0049 (6)
C2	0.0219 (7)	0.0273 (7)	0.0349 (8)	0.0015 (6)	−0.0020 (6)	−0.0129 (6)
C3	0.0227 (7)	0.0279 (7)	0.0223 (6)	0.0052 (6)	0.0015 (5)	−0.0055 (5)
C4	0.0183 (6)	0.0220 (6)	0.0185 (6)	0.0042 (5)	0.0015 (5)	−0.0022 (5)
C5	0.0210 (6)	0.0229 (6)	0.0180 (6)	0.0061 (5)	0.0026 (5)	−0.0006 (5)
C6	0.0203 (6)	0.0253 (7)	0.0234 (6)	−0.0005 (6)	0.0038 (5)	0.0070 (5)
C7	0.0185 (6)	0.0182 (6)	0.0204 (6)	0.0001 (5)	0.0031 (5)	0.0018 (5)
C8	0.0167 (6)	0.0178 (6)	0.0188 (6)	0.0023 (5)	0.0010 (4)	−0.0002 (5)
C9	0.0181 (6)	0.0218 (6)	0.0191 (6)	−0.0008 (5)	0.0010 (5)	−0.0009 (5)
C10	0.0216 (6)	0.0239 (7)	0.0218 (6)	−0.0010 (5)	0.0049 (5)	0.0010 (5)
C11	0.0293 (7)	0.0281 (7)	0.0171 (6)	0.0017 (6)	0.0042 (5)	−0.0019 (5)
C12	0.0335 (8)	0.0240 (7)	0.0218 (6)	−0.0036 (6)	0.0006 (5)	−0.0061 (5)
N1	0.0189 (5)	0.0237 (6)	0.0158 (5)	−0.0006 (4)	0.0024 (4)	0.0035 (4)
N2	0.0175 (5)	0.0200 (5)	0.0155 (5)	0.0018 (4)	0.0024 (4)	0.0020 (4)
N3	0.0281 (6)	0.0200 (6)	0.0218 (5)	−0.0030 (5)	0.0008 (4)	−0.0022 (4)
O1	0.0378 (6)	0.0339 (6)	0.0161 (4)	0.0009 (5)	0.0051 (4)	0.0002 (4)
S1	0.02249 (19)	0.02627 (19)	0.02056 (18)	−0.00532 (13)	0.00245 (12)	−0.00153 (12)

### N-Methyl-N'-[(E)-pyridin-2-ylmethylidene]thiophene-2-carbohydrazide (III) Geometric parameters (Å, º)

**Table:**

C1—C2	1.359 (2)	C7—N2	1.2815 (17)
C1—S1	1.7075 (14)	C7—C8	1.4709 (17)
C1—H1	0.9500	C7—H7	0.9500
C2—C3	1.414 (2)	C8—N3	1.3436 (17)
C2—H2	0.9500	C8—C9	1.3981 (18)
C3—C4	1.3894 (19)	C9—C10	1.3879 (18)
C3—H3	0.9500	C9—H9	0.9500
C4—C5	1.4817 (19)	C10—C11	1.3835 (19)
C4—S1	1.7223 (13)	C10—H10	0.9500
C5—O1	1.2225 (16)	C11—C12	1.382 (2)
C5—N1	1.3780 (17)	C11—H11	0.9500
C6—N1	1.4545 (17)	C12—N3	1.3400 (17)
C6—H6A	0.9800	C12—H12	0.9500
C6—H6B	0.9800	N1—N2	1.3690 (14)
C6—H6C	0.9800		
			
C2—C1—S1	112.41 (11)	C8—C7—H7	119.5
C2—C1—H1	123.8	N3—C8—C9	123.07 (11)
S1—C1—H1	123.8	N3—C8—C7	113.86 (11)
C1—C2—C3	112.48 (13)	C9—C8—C7	123.06 (11)
C1—C2—H2	123.8	C10—C9—C8	118.36 (12)
C3—C2—H2	123.8	C10—C9—H9	120.8
C4—C3—C2	112.53 (12)	C8—C9—H9	120.8
C4—C3—H3	123.7	C11—C10—C9	118.95 (13)
C2—C3—H3	123.7	C11—C10—H10	120.5
C3—C4—C5	120.19 (12)	C9—C10—H10	120.5
C3—C4—S1	110.61 (10)	C12—C11—C10	118.69 (12)
C5—C4—S1	129.19 (10)	C12—C11—H11	120.7
O1—C5—N1	120.00 (13)	C10—C11—H11	120.7
O1—C5—C4	119.42 (12)	N3—C12—C11	123.71 (13)
N1—C5—C4	120.58 (11)	N3—C12—H12	118.1
N1—C6—H6A	109.5	C11—C12—H12	118.1
N1—C6—H6B	109.5	N2—N1—C5	118.24 (11)
H6A—C6—H6B	109.5	N2—N1—C6	121.81 (11)
N1—C6—H6C	109.5	C5—N1—C6	119.89 (11)
H6A—C6—H6C	109.5	C7—N2—N1	118.14 (11)
H6B—C6—H6C	109.5	C12—N3—C8	117.20 (12)
N2—C7—C8	121.06 (12)	C1—S1—C4	91.97 (7)
N2—C7—H7	119.5		
			
S1—C1—C2—C3	−0.08 (16)	C10—C11—C12—N3	0.7 (2)
C1—C2—C3—C4	0.02 (18)	O1—C5—N1—N2	178.23 (11)
C2—C3—C4—C5	−179.06 (12)	C4—C5—N1—N2	−1.19 (18)
C2—C3—C4—S1	0.04 (15)	O1—C5—N1—C6	1.06 (19)
C3—C4—C5—O1	−2.21 (19)	C4—C5—N1—C6	−178.37 (11)
S1—C4—C5—O1	178.88 (10)	C8—C7—N2—N1	−179.79 (11)
C3—C4—C5—N1	177.22 (12)	C5—N1—N2—C7	179.40 (12)
S1—C4—C5—N1	−1.69 (19)	C6—N1—N2—C7	−3.49 (18)
N2—C7—C8—N3	176.35 (12)	C11—C12—N3—C8	−1.0 (2)
N2—C7—C8—C9	−4.8 (2)	C9—C8—N3—C12	0.5 (2)
N3—C8—C9—C10	0.3 (2)	C7—C8—N3—C12	179.38 (12)
C7—C8—C9—C10	−178.51 (12)	C2—C1—S1—C4	0.09 (12)
C8—C9—C10—C11	−0.6 (2)	C3—C4—S1—C1	−0.07 (11)
C9—C10—C11—C12	0.1 (2)	C5—C4—S1—C1	178.92 (13)

### N-Methyl-N'-[(E)-pyridin-2-ylmethylidene]thiophene-2-carbohydrazide (III) Hydrogen-bond geometry (Å, º)

**Table:**

*D*—H···*A*	*D*—H	H···*A*	*D*···*A*	*D*—H···*A*
C9—H9···S1	0.95	2.84	3.7217 (13)	155
C6—H6*C*···N3^i^	0.98	2.61	3.3499 (18)	132

Symmetry code: (i) −*x*+1/2, *y*+1/2, −*z*+1/2.

### N'-[(E)-Pyridin-2-ylmethylidene]-2-(thiophen-2-yl)ethanohydrazide (IV) Crystal data

**Table:**

C_12_H_11_N_3_OS	*F*(000) = 512
*M**_r_* = 245.30	*D*_x_ = 1.414 Mg m^−^^3^
Monoclinic, *P*2_1_/*c*	Mo *K*α radiation, λ = 0.71073 Å
*a* = 11.3963 (8) Å	Cell parameters from 43879 reflections
*b* = 9.2782 (7) Å	θ = 2.9–27.5°
*c* = 11.8178 (8) Å	µ = 0.27 mm^−^^1^
β = 112.761 (2)°	*T* = 100 K
*V* = 1152.27 (14) Å^3^	Block, colourless
*Z* = 4	0.10 × 0.09 × 0.06 mm

### N'-[(E)-Pyridin-2-ylmethylidene]-2-(thiophen-2-yl)ethanohydrazide (IV) Data collection

**Table:**

Rigaku AFC12 CCD diffractometer	2138 reflections with *I* > 2σ(*I*)
ω scans	*R*_int_ = 0.031
Absorption correction: multi-scan (CrystalClear; Rigaku, 2012)	θ_max_ = 28.4°, θ_min_ = 2.9°
*T*_min_ = 0.723, *T*_max_ = 1.000	*h* = −15→14
8155 measured reflections	*k* = −12→11
2593 independent reflections	*l* = −14→14

### N'-[(E)-Pyridin-2-ylmethylidene]-2-(thiophen-2-yl)ethanohydrazide (IV) Refinement

**Table:**

Refinement on *F*^2^	Primary atom site location: structure-invariant direct methods
Least-squares matrix: full	Hydrogen site location: mixed
*R*[*F*^2^ > 2σ(*F*^2^)] = 0.041	H atoms treated by a mixture of independent and constrained refinement
*wR*(*F*^2^) = 0.112	*w* = 1/[σ^2^(*F*_o_^2^) + (0.0505*P*)^2^ + 0.7045*P*] where *P* = (*F*_o_^2^ + 2*F*_c_^2^)/3
*S* = 1.10	(Δ/σ)_max_ < 0.001
2593 reflections	Δρ_max_ = 0.32 e Å^−^^3^
170 parameters	Δρ_min_ = −0.30 e Å^−^^3^
10 restraints	

### N'-[(E)-Pyridin-2-ylmethylidene]-2-(thiophen-2-yl)ethanohydrazide (IV) Special details

**Table:**

Geometry. All esds (except the esd in the dihedral angle between two l.s. planes) are estimated using the full covariance matrix. The cell esds are taken into account individually in the estimation of esds in distances, angles and torsion angles; correlations between esds in cell parameters are only used when they are defined by crystal symmetry. An approximate (isotropic) treatment of cell esds is used for estimating esds involving l.s. planes.

### N'-[(E)-Pyridin-2-ylmethylidene]-2-(thiophen-2-yl)ethanohydrazide (IV) Fractional atomic coordinates and isotropic or equivalent isotropic displacement parameters (Å^2^)

**Table:**

	*x*	*y*	*z*	*U*_iso_*/*U*_eq_	Occ. (<1)
C1	0.9466 (2)	0.7136 (3)	0.2749 (2)	0.0222 (5)	0.851 (2)
H1	1.033688	0.689483	0.317920	0.027*	0.851 (2)
C2	0.8750 (2)	0.6617 (3)	0.1571 (2)	0.0203 (5)	0.851 (2)
H2	0.905946	0.598307	0.111806	0.024*	0.851 (2)
C3	0.7527 (8)	0.7158 (11)	0.1161 (7)	0.0246 (10)	0.851 (2)
H3	0.689801	0.695835	0.037087	0.029*	0.851 (2)
C4	0.73066 (15)	0.80326 (17)	0.20255 (15)	0.0211 (3)	0.851 (2)
S1	0.86521 (5)	0.82422 (7)	0.33564 (5)	0.02360 (18)	0.851 (2)
C1B	0.8982 (14)	0.684 (2)	0.1833 (17)	0.0203 (5)	0.149 (2)
H1B	0.956589	0.632055	0.158977	0.024*	0.149 (2)
C2B	0.9327 (13)	0.7537 (17)	0.2944 (14)	0.0222 (5)	0.149 (2)
H2B	1.015819	0.750408	0.356806	0.027*	0.149 (2)
C3B	0.8327 (13)	0.829 (2)	0.3049 (14)	0.02360 (18)	0.149 (2)
H3B	0.836035	0.888345	0.371821	0.028*	0.149 (2)
C4B	0.73066 (15)	0.80326 (17)	0.20255 (15)	0.0211 (3)	0.149 (2)
S1B	0.7385 (12)	0.6996 (17)	0.0929 (12)	0.0246 (10)	0.149 (2)
C5	0.61018 (15)	0.86577 (18)	0.19459 (16)	0.0231 (3)	
H5A	0.553207	0.878920	0.107425	0.028*	
H5B	0.625573	0.961417	0.234861	0.028*	
C6	0.54702 (14)	0.76587 (17)	0.25759 (15)	0.0206 (3)	
C7	0.35889 (15)	0.83579 (18)	0.42527 (15)	0.0223 (3)	
H7	0.359384	0.937962	0.420223	0.027*	
C8	0.28771 (14)	0.76417 (18)	0.49016 (15)	0.0207 (3)	
C9	0.27577 (15)	0.61405 (19)	0.49162 (16)	0.0243 (4)	
H9	0.314773	0.554343	0.450832	0.029*	
C10	0.20629 (15)	0.55382 (19)	0.55336 (16)	0.0271 (4)	
H10	0.197174	0.452251	0.556146	0.033*	
C11	0.14999 (16)	0.64555 (19)	0.61139 (16)	0.0260 (4)	
H11	0.101007	0.607862	0.653853	0.031*	
C12	0.16714 (16)	0.79323 (19)	0.60568 (17)	0.0267 (4)	
H12	0.129255	0.854810	0.646226	0.032*	
N1	0.48282 (13)	0.83765 (15)	0.31638 (13)	0.0211 (3)	
H1N	0.4714 (18)	0.932 (2)	0.3080 (18)	0.025*	
N2	0.42014 (12)	0.76009 (15)	0.37563 (13)	0.0213 (3)	
N3	0.23384 (13)	0.85429 (16)	0.54651 (13)	0.0247 (3)	
O1	0.55355 (11)	0.63460 (13)	0.25449 (13)	0.0280 (3)	

### N'-[(E)-Pyridin-2-ylmethylidene]-2-(thiophen-2-yl)ethanohydrazide (IV) Atomic displacement parameters (Å^2^)

**Table:**

	*U*^11^	*U*^22^	*U*^33^	*U*^12^	*U*^13^	*U*^23^
C1	0.0172 (8)	0.0271 (13)	0.0254 (12)	0.0030 (8)	0.0118 (7)	0.0006 (9)
C2	0.0203 (11)	0.0209 (13)	0.0221 (14)	−0.0002 (9)	0.0109 (10)	−0.0036 (8)
C3	0.0205 (18)	0.024 (2)	0.028 (3)	−0.0032 (14)	0.0080 (19)	−0.0029 (17)
C4	0.0196 (7)	0.0194 (7)	0.0245 (8)	−0.0023 (6)	0.0088 (6)	0.0032 (6)
S1	0.0191 (3)	0.0280 (3)	0.0230 (3)	−0.0014 (2)	0.0073 (2)	−0.0037 (2)
C1B	0.0203 (11)	0.0209 (13)	0.0221 (14)	−0.0002 (9)	0.0109 (10)	−0.0036 (8)
C2B	0.0172 (8)	0.0271 (13)	0.0254 (12)	0.0030 (8)	0.0118 (7)	0.0006 (9)
C3B	0.0191 (3)	0.0280 (3)	0.0230 (3)	−0.0014 (2)	0.0073 (2)	−0.0037 (2)
C4B	0.0196 (7)	0.0194 (7)	0.0245 (8)	−0.0023 (6)	0.0088 (6)	0.0032 (6)
S1B	0.0205 (18)	0.024 (2)	0.028 (3)	−0.0032 (14)	0.0080 (19)	−0.0029 (17)
C5	0.0204 (7)	0.0176 (7)	0.0319 (9)	−0.0002 (6)	0.0108 (6)	0.0024 (7)
C6	0.0164 (6)	0.0194 (8)	0.0244 (8)	−0.0001 (6)	0.0061 (6)	0.0011 (6)
C7	0.0219 (7)	0.0192 (8)	0.0251 (8)	0.0000 (6)	0.0083 (6)	0.0006 (6)
C8	0.0181 (7)	0.0218 (8)	0.0211 (8)	0.0005 (6)	0.0064 (6)	−0.0013 (6)
C9	0.0212 (7)	0.0231 (8)	0.0298 (9)	0.0009 (6)	0.0112 (6)	−0.0033 (7)
C10	0.0224 (7)	0.0222 (8)	0.0354 (10)	−0.0002 (6)	0.0098 (7)	0.0021 (7)
C11	0.0239 (7)	0.0267 (9)	0.0286 (9)	−0.0009 (6)	0.0114 (7)	0.0032 (7)
C12	0.0273 (8)	0.0277 (9)	0.0300 (9)	0.0014 (7)	0.0163 (7)	−0.0025 (7)
N1	0.0213 (6)	0.0168 (6)	0.0271 (7)	0.0006 (5)	0.0114 (6)	0.0015 (5)
N2	0.0183 (6)	0.0211 (7)	0.0244 (7)	−0.0007 (5)	0.0081 (5)	0.0013 (5)
N3	0.0247 (6)	0.0225 (7)	0.0290 (8)	0.0012 (5)	0.0128 (6)	−0.0003 (6)
O1	0.0267 (6)	0.0161 (6)	0.0475 (8)	−0.0002 (5)	0.0214 (6)	0.0000 (5)

### N'-[(E)-Pyridin-2-ylmethylidene]-2-(thiophen-2-yl)ethanohydrazide (IV) Geometric parameters (Å, º)

**Table:**

C1—C2	1.399 (3)	C5—H5A	0.9900
C1—S1	1.716 (2)	C5—H5B	0.9900
C1—H1	0.9500	C6—O1	1.222 (2)
C2—C3	1.380 (8)	C6—N1	1.362 (2)
C2—H2	0.9500	C7—N2	1.281 (2)
C3—C4	1.401 (7)	C7—C8	1.473 (2)
C3—H3	0.9500	C7—H7	0.9500
C4—C5	1.460 (2)	C8—N3	1.355 (2)
C4—S1	1.7311 (16)	C8—C9	1.400 (2)
C1B—C2B	1.379 (15)	C9—C10	1.385 (2)
C1B—S1B	1.723 (16)	C9—H9	0.9500
C1B—H1B	0.9500	C10—C11	1.395 (2)
C2B—C3B	1.384 (15)	C10—H10	0.9500
C2B—H2B	0.9500	C11—C12	1.389 (2)
C3B—C4B	1.337 (13)	C11—H11	0.9500
C3B—H3B	0.9500	C12—N3	1.341 (2)
C4B—C5	1.460 (2)	C12—H12	0.9500
C4B—S1B	1.643 (11)	N1—N2	1.3804 (19)
C5—C6	1.532 (2)	N1—H1N	0.88 (2)
			
C2—C1—S1	114.85 (18)	C4—C5—H5B	109.8
C2—C1—H1	122.6	C6—C5—H5B	109.8
S1—C1—H1	122.6	H5A—C5—H5B	108.2
C3—C2—C1	110.2 (3)	O1—C6—N1	123.63 (15)
C3—C2—H2	124.9	O1—C6—C5	122.90 (15)
C1—C2—H2	124.9	N1—C6—C5	113.47 (14)
C2—C3—C4	113.4 (5)	N2—C7—C8	119.90 (15)
C2—C3—H3	123.3	N2—C7—H7	120.1
C4—C3—H3	123.3	C8—C7—H7	120.1
C3—C4—C5	127.8 (3)	N3—C8—C9	122.90 (15)
C3—C4—S1	112.4 (3)	N3—C8—C7	115.03 (15)
C5—C4—S1	119.63 (13)	C9—C8—C7	122.07 (15)
C1—S1—C4	89.14 (10)	C10—C9—C8	119.07 (16)
C2B—C1B—S1B	113.1 (12)	C10—C9—H9	120.5
C2B—C1B—H1B	123.4	C8—C9—H9	120.5
S1B—C1B—H1B	123.4	C9—C10—C11	118.56 (16)
C1B—C2B—C3B	112.6 (13)	C9—C10—H10	120.7
C1B—C2B—H2B	123.7	C11—C10—H10	120.7
C3B—C2B—H2B	123.7	C12—C11—C10	118.53 (16)
C4B—C3B—C2B	106.5 (12)	C12—C11—H11	120.7
C4B—C3B—H3B	126.7	C10—C11—H11	120.7
C2B—C3B—H3B	126.7	N3—C12—C11	124.09 (16)
C3B—C4B—C5	117.0 (7)	N3—C12—H12	118.0
C3B—C4B—S1B	121.7 (7)	C11—C12—H12	118.0
C5—C4B—S1B	121.2 (4)	C6—N1—N2	119.30 (13)
C4B—S1B—C1B	85.9 (8)	C6—N1—H1N	120.7 (13)
C4B—C5—C6	109.60 (13)	N2—N1—H1N	119.4 (13)
C4—C5—C6	109.60 (13)	C7—N2—N1	115.27 (14)
C4—C5—H5A	109.8	C12—N3—C8	116.84 (15)
C6—C5—H5A	109.8		
			
S1—C1—C2—C3	1.1 (6)	C4B—C5—C6—O1	−35.6 (2)
C1—C2—C3—C4	−2.0 (9)	C4—C5—C6—O1	−35.6 (2)
C2—C3—C4—C5	−173.4 (4)	C4B—C5—C6—N1	144.72 (14)
C2—C3—C4—S1	2.1 (9)	C4—C5—C6—N1	144.72 (14)
C2—C1—S1—C4	0.1 (2)	N2—C7—C8—N3	175.58 (14)
C3—C4—S1—C1	−1.2 (5)	N2—C7—C8—C9	−5.2 (2)
C5—C4—S1—C1	174.74 (15)	N3—C8—C9—C10	−0.2 (2)
S1B—C1B—C2B—C3B	−4 (2)	C7—C8—C9—C10	−179.31 (15)
C1B—C2B—C3B—C4B	3 (2)	C8—C9—C10—C11	0.4 (2)
C2B—C3B—C4B—C5	176.8 (10)	C9—C10—C11—C12	−0.6 (2)
C2B—C3B—C4B—S1B	−0.5 (19)	C10—C11—C12—N3	0.7 (3)
C3B—C4B—S1B—C1B	−1.5 (16)	O1—C6—N1—N2	−0.5 (2)
C5—C4B—S1B—C1B	−178.7 (8)	C5—C6—N1—N2	179.18 (13)
C2B—C1B—S1B—C4B	3.1 (18)	C8—C7—N2—N1	179.54 (13)
C3B—C4B—C5—C6	−84.9 (10)	C6—N1—N2—C7	−177.90 (14)
S1B—C4B—C5—C6	92.4 (7)	C11—C12—N3—C8	−0.5 (3)
C3—C4—C5—C6	93.8 (6)	C9—C8—N3—C12	0.2 (2)
S1—C4—C5—C6	−81.44 (16)	C7—C8—N3—C12	179.39 (14)

### N'-[(E)-Pyridin-2-ylmethylidene]-2-(thiophen-2-yl)ethanohydrazide (IV) Hydrogen-bond geometry (Å, º)

**Table:**

*D*—H···*A*	*D*—H	H···*A*	*D*···*A*	*D*—H···*A*
N1—H1*N*···O1^i^	0.88 (2)	2.00 (2)	2.8628 (18)	164.9 (18)
C3—H3···N1^ii^	0.95	2.78	3.718 (7)	172
C5—H5*B*···O1^i^	0.99	2.64	3.307 (2)	125
C7—H7···S1*B*^i^	0.95	2.65	3.534 (16)	155
C12—H12···S1^iii^	0.95	2.98	3.6624 (19)	129

Symmetry codes: (i) −*x*+1, *y*+1/2, −*z*+1/2; (ii) *x*, −*y*+3/2, *z*−1/2; (iii) −*x*+1, −*y*+2, −*z*+1.
