# Supplementary material for: Different conformations and packing motifs in the crystal structures of four thio­phene–carbohydrazide–pyridine derivatives
Source: Acta Crystallogr E Crystallogr Commun. 2022 May 17;78(Pt 6):619–24. doi: 10.1107/S2056989022005151 (PMC9431791; doi:10.1107/S2056989022005151)
Supplement: Supplementary file 10 [file e-78-00619-sup10.docx]

**Supplementary figure: Hirshfeld surfaces for (I)–(IV)**

**
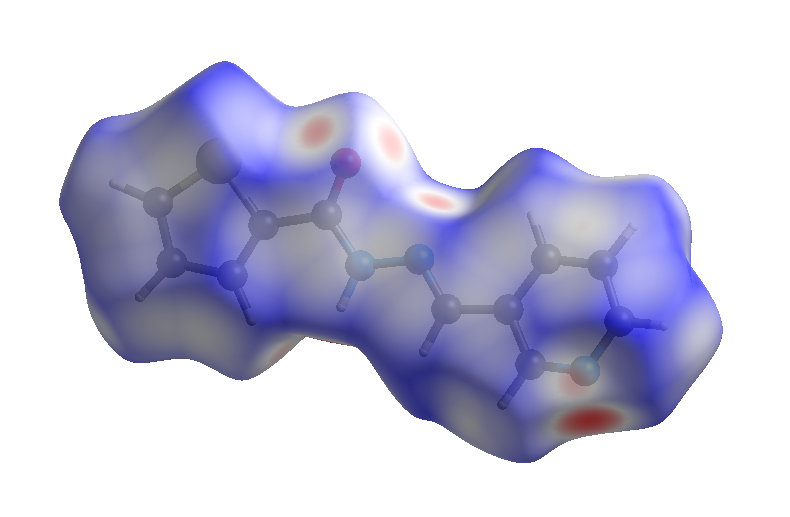
**

**Hirshfeld surface of (I)**

**
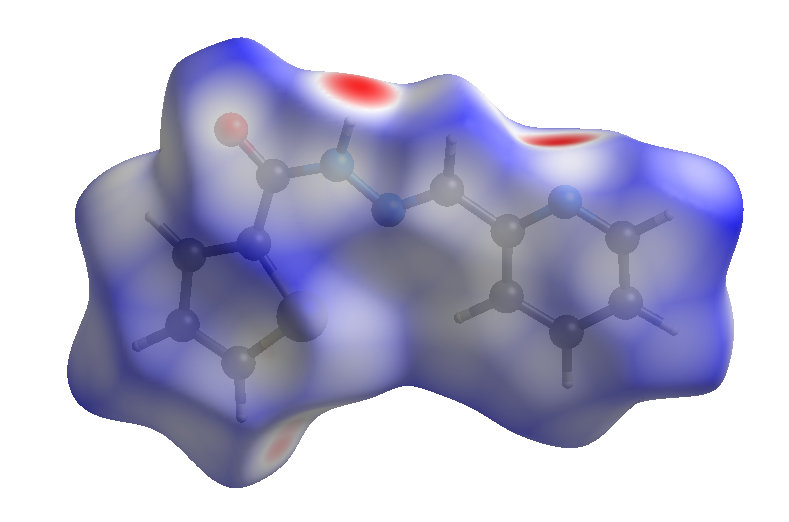
**

**Hirshfeld surface of (II)**

**
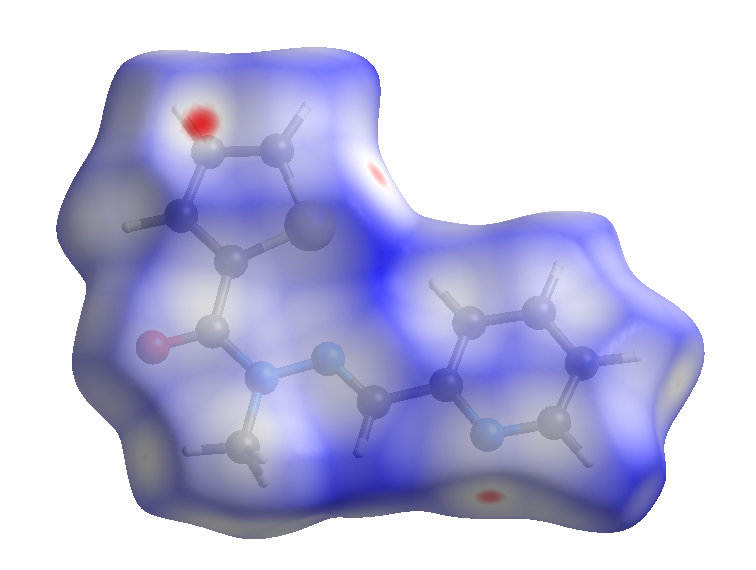
**

**Hirshfeld surface of (III)**

**
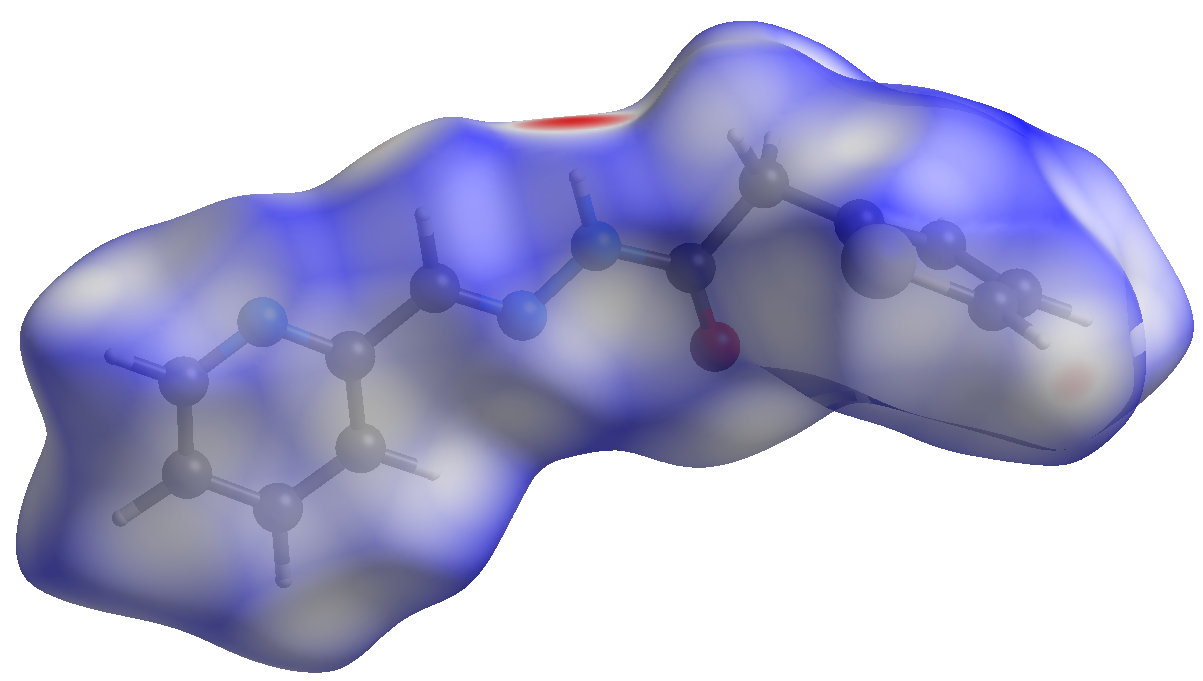
**

**Hirshfeld surface of (IV)**
